# Urine-derived induced pluripotent stem cells as a modeling tool for paroxysmal kinesigenic dyskinesia

**DOI:** 10.1242/bio.013078

**Published:** 2015-11-30

**Authors:** Shu-Zhen Zhang, Hong-Fu Li, Li-Xiang Ma, Wen-Jing Qian, Zhong-Feng Wang, Zhi-Ying Wu

**Affiliations:** 1 Department of Neurology andInstitute of Neurology, Huashan Hospital, Institutes of Brain Science and State Key Laboratory of Medical Neurobiology, Shanghai Medical College, Fudan University, Shanghai 200040, China; 2 Department of Neurology andResearch Center of Neurology in Second Affiliated Hospital, and the Collaborative Innovation Center for Brain Science, Zhejiang University School of Medicine, Hangzhou 310009, China; 3 Department of Anatomy, Histology & Embryology, Shanghai Medical College, Fudan University, Shanghai 200032, China; 4 Institutes of Brain Science, Institute of Neurobiology and State Key Laboratory of Medical Neurobiology, Fudan University, Shanghai 200032, China

**Keywords:** Paroxysmal kinesigenic dyskinesia, Proline-rich transmembrane protein 2, Human induced pluripotent stem cells

## Abstract

Paroxysmal kinesigenic dyskinesia (PKD) is a monogenic movement disorder with autosomal dominant inheritance. We previously identified the proline-rich transmembrane protein 2 (*PRRT2*) as a causative gene of PKD. However, the pathogenesis of PKD remains largely unknown so far. In addition, applicable modeling tools to investigate the underlying mechanisms of PKD are still lacking. The combination of disease-specific human induced pluripotent stem cells (iPSCs) and directed cell differentiation offers an ideal platform for disease modeling. In this study, we generated two iPSC lines from the renal epithelial cells of one PKD patient with the hotspot c.649dupC mutation (PKD-iPSCs). These cell lines were positive for alkaline phosphatase Nanog, Tra-1-80, Tra-1-60, SSEA-3 and SSEA-4. Teratomas with three blastoderms including ectoderm, mesoderm, and endoderm were obtained two months after injection of PKD-iPSCs into NOD/SCID mice. The expression of *PRRT2* mRNA was decreased in PKD-iPSCs compared with that of the control iPSCs. Furthermore, PKD-iPSCs possessed the differentiation potential of functional glutamatergic, dopaminergic and motor neurons *in vitro*. Electrophysiological examinations revealed that the current densities of fast activated and deactivated sodium channels as well as voltage gated potassium channels were not different between the neurons from PKD-iPSCs and control iPSCs. Thus, PKD-iPSCs are a feasible modeling tool to investigate the pathogenic mechanisms of PKD.

## Abstract (precis)

**Summary:** This is the first report of urinary cell-induced pluripotent stem cells being used as resources for investigation of the pathological mechanisms of paroxysmal kinesigenic dyskinesia.

## INTRODUCTION

Paroxysmal kinesigenic dyskinesia (PKD) is a common subgroup of the paroxysmal dyskinesias and is characterized by short episodes of involuntary movement attacks triggered by sudden movements (Bruno et al., 2004). It is inherited in an autosomal dominant pattern with an incomplete penetrance. We initially reported the proline-rich transmembrane protein 2 gene (*PRRT2*) as a causative gene of PKD (Chen et al., 2011), which was subsequently confirmed by isolated groups (Li et al., 2012; Wang et al., 2011; Lee et al., 2012a). To date more than 50 PRRT2 mutations have been identified world-wide, among which c.649dupC (p.R217Pfs*8) is a mutation hotspot (Heron and Dibbens, 2013), and could be derived from *de novo* in PKD patients (Li et al., 2013a).

Further investigations have shown that infantile convulsions with choreoathetosis (ICCA) syndrome (Cai et al., 2012; Heron et al., 2012; Lee et al., 2012b) and benign familial infantile epilepsy (BFIE) (de Vries et al., 2012; Heron et al., 2012) are also induced by *PRRT2* mutations. It has been found that the same hotspot mutation in *PRRT2* can cause BFIE alone, PKD alone, or both of them (Heron and Dibbens, 2013). This suggests that the pathology caused by *PRRT2* mutations is influenced by other genetic or environmental factors. These additional factors are currently unknown.

PRRT2 is a largely uncharacterized protein. Until now, only three groups have explored its possible functions (Chen et al., 2011; Heron et al., 2012; Lee et al., 2012a). It is highly expressed in the brain (Chen et al., 2011; Heron et al., 2012) and has been shown to interact with SNAP-25 (Lee et al., 2012a). However, the potential mechanisms underlying PKD remains unknown so far. There are several reasons why investigations have been hampered. First, *PRRT2* is a newly reported gene with little-known physiological functions. Second, a low-dose of carbamazepine can completely control the attacks in cases with *PRRT2* mutation (Li et al., 2013b). Third, interictal neurological examinations are normal and the ictal electroencephalograph (EEG) is usually uninformative (van Rootselaar et al., 2009). Therefore, brain biopsy tissues from PKD patients to investigate the physiological role of PRRT2 are rarely obtained. In addition, applicable modeling tools to investigate the underlying mechanisms of PKD are still lacking.

Recently, the technology of reprogramming of somatic cells to a pluripotent state emerged (Takahashi and Yamanaka, 2006; Takahashi et al., 2007). The combination of disease-specific human induced pluripotent stem cells (iPSC) and directed cell differentiation offers an ideal platform for modeling and studying many human diseases (Allodi and Hedlund, 2014; Imaizumi and Okano, 2014; Isobe et al., 2014).

In this study, for the first time, we generated iPSC lines from the urine of one PKD patient with the p.R217Pfs*8 mutation. These patient-specific iPSCs possessed an expression signature similar to human ES cells (hESCs) and can be differentiated into the cell types that represent each of the three embryonic germ layers. The PKD-iPSCs were capable of producing phenotypically normal, functional glutamatergic, motor and dopaminergic neurons. But the frequency and amplitude of fast activated and deactivated sodium channels as well as voltage-gated potassium channels of PKD-iPSC-induced neurons showed no differences compared with those of control (CON)-iPSC-induced neurons. Our cells represent a promising modeling tool for the investigation of the pathogenesis of PKD.

## RESULTS

### Generation and characterization of iPSCs

**Fig. 1.**
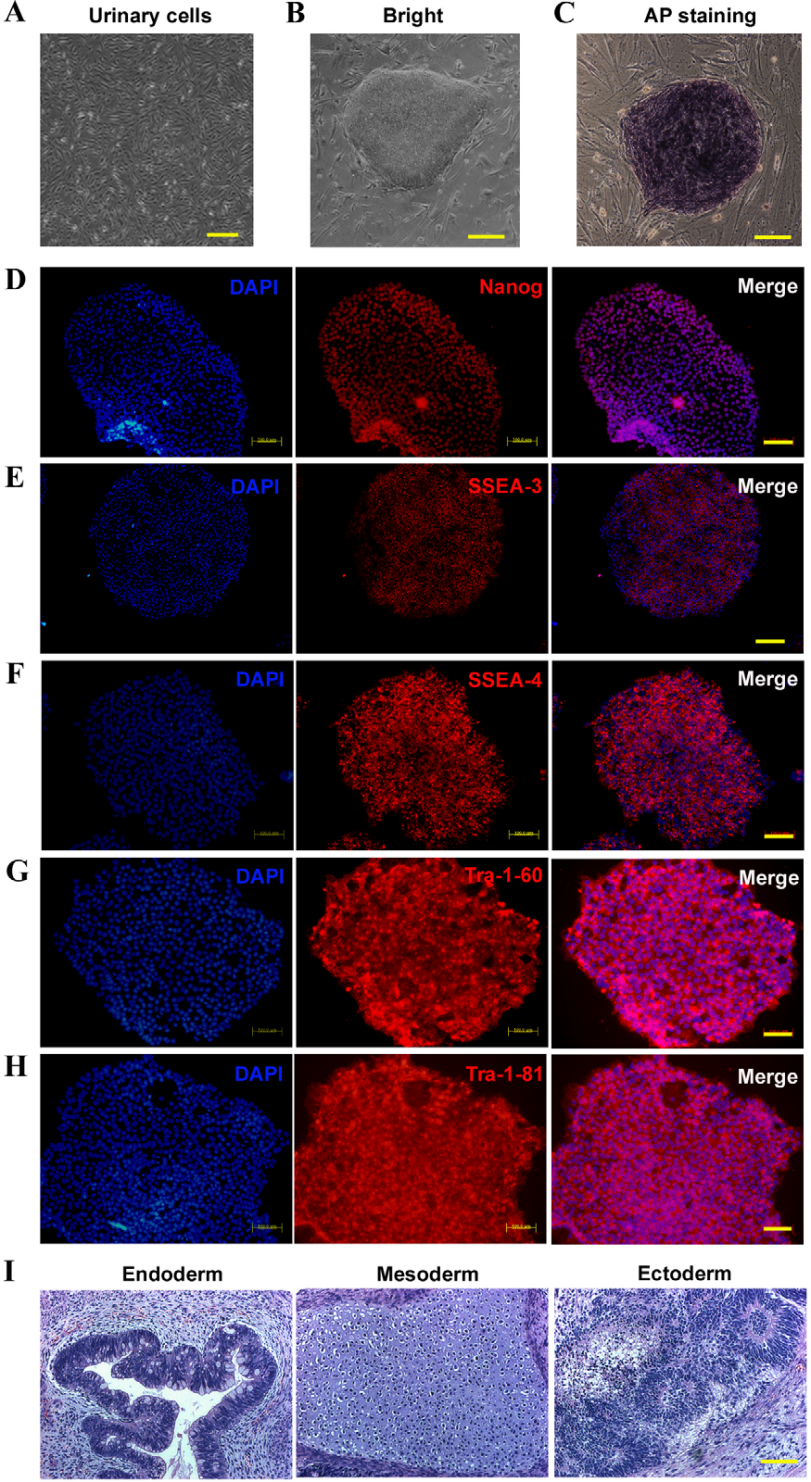
**Characterization of iPSCs derived from the PKD patient.** (A) Urinary cells were collected from the PKD patient carrying the c.649dupC mutation. (B) PKD-iPS cell lines were induced from the urinary cells. (C) PKD-iPS cell lines were positive for AP. (D-H) PKD-iPS cell lines were positive for pluripotency markers including Nanog (D), SSEA-3 (E), SSEA-4 (F), Tra-1-60 (G), and Tra-1-81 (H). (I) H&E staining images of teratomas formed by iPSCs injected into adult NOD/SCID mice. Scale bars: 200 μm.

According to a previously described protocol, primary urine cells (Fig. 1A) were collected from one PKD patient with the *PRRT2* c.649dupC mutation (Zhou et al., 2012). Cells were infected with retroviruses encoding Oct4, Sox2, Klf4 and c-Myc to generate iPSCs (Takahashi et al., 2007). Two clones were obtained. The morphology exhibited by the iPSC colonies was similar to those of human ES cells (Fig. 1B) and the colonies were positive for alkaline phosphatase (Fig. 1C). These iPSCs expressed endogenous pluripotency markers Nanog, Tra-1-80, Tra-1-60, SSEA-3 and SSEA-4, demonstrated as the immunofluorescence (Fig. 1D-H). The fully reprogrammed iPSCs formed teratomas in NOD/SCID mice (non-obese diabetes/severe combined immunodeficient mice, an ideal model for tumor biology and xenograft research) 8 weeks after injection. The endoderm (glandular structures), mesoderm (cartilage), and ectoderm (pigmented epithelium) were each detected in the teratomas formed (Fig. 1I). These results suggest that PKD-iPSCs can spontaneously differentiate into derivatives of all three germ layers *in vivo*.

### Karyotyping and genotyping of control and PKD-iPSCs

**Fig. 2.**
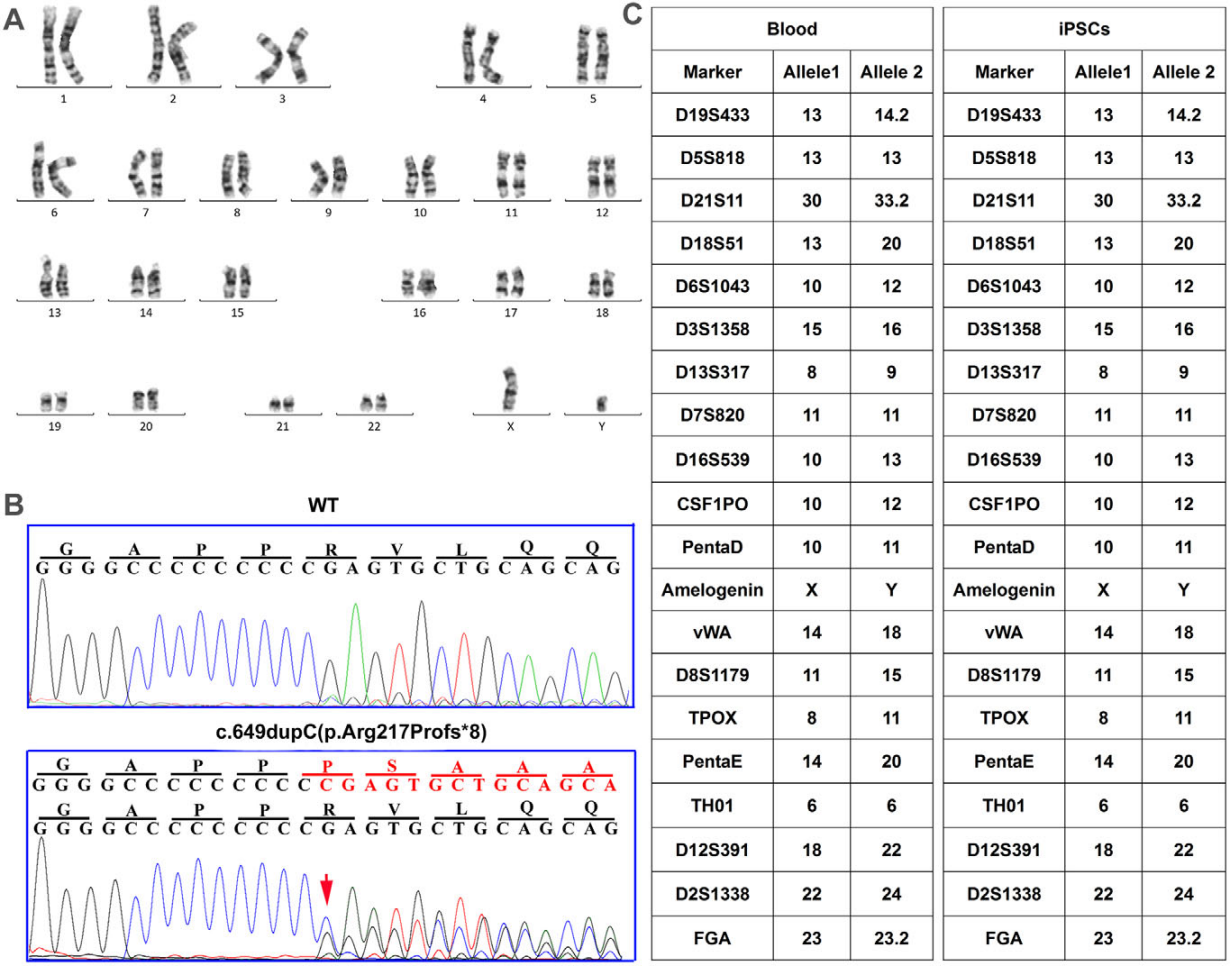
**Karyotyping and genotyping of iPSCs derived from the PKD patient.** (A) The iPSCs derived from the PKD patient have normal karyotype. (B) The iPS colonies derived from urinary cells of the PKD patient were verified to carry the heterozygous c.649dupC (p.Arg217Profs*8) mutation via Sanger sequencing. (C) Short tandem repeats and Amelogenin analyses were performed on the patient's blood and iPSCs.

The karyotype of the generated iPSC line was analyzed, which showed normal karyotype (Fig. 2A). Sanger sequencing revealed that the PKD-iPSCs carried the PRRT2 c.649dupC mutation (Fig. 2B). In addition, short tandem repeat analysis between patient blood sample of the patient and PKD-iPSCs were performed. The results were matched, verifying that the PKD-iPSC lines were genetically derived from the patient (Fig. 2C).

### Expression of *PRRT2* mRNA in iPSCs

**Fig. 3.**
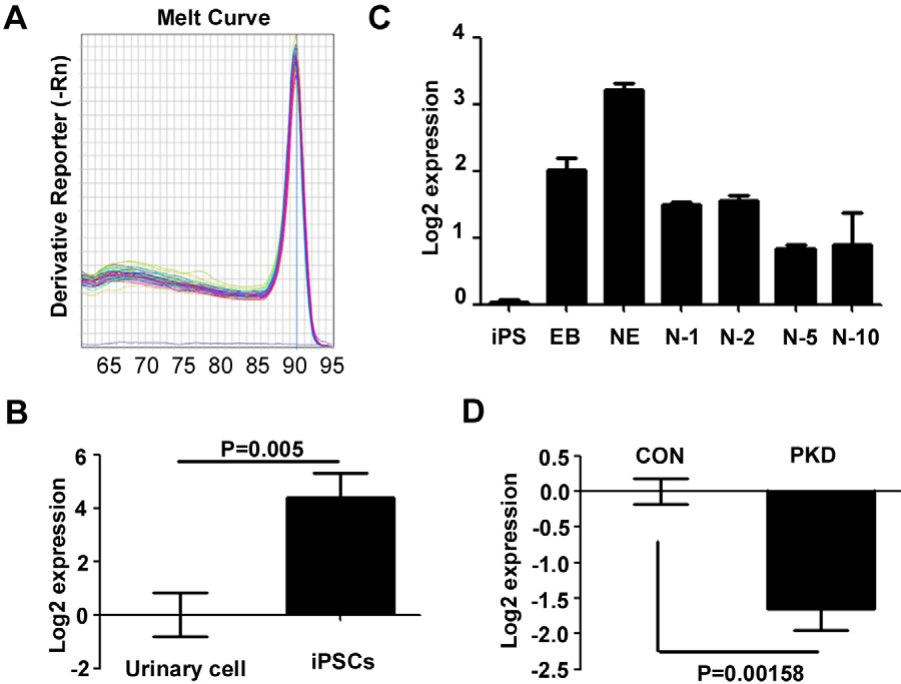
**Expression pattern of *PRRT2* in iPSCs.** Relative quantitative PCR was used to measure the *PRRT2* mRNA expression. Values are normalized to the median of each control group and plotted in log2 scale. (A) Melting curve of the *PRRT2* primers. (B) The iPSCs expressed more *PRRT2* than primary urine cells. Student's *t*-test was used to analyze the expression difference of *PRRT2* between iPSCs and urine cell group. *N*=6, *P*<0.01 (C) The expression of *PRRT2* mRNA peaked in the neuroepithelial stage during the differentiation from iPSCs into neurons. EB, embryonic body; NE, neuroepithelial cells; N-x, x day after replacing on glasses. One way ANOVA was used to analyze whether the time factor would influence the expression of PRRT2. Friedman value is 16.86, *P*=0.0098. (D) The PKD-iPSCs expressed less *PRRT2* than CON-iPSCs. Student's *t*-test was used to test whether there is significant difference between the CON and PKD group. Error bars represent s.e.m.

iPSCs offer an unprecedented opportunity to model human disease. However, it is unclear if the obtained iPSCs could express *PRRT2*. We thus investigated the *PRRT2* mRNA in urine cells and induced CON-iPSCs. The melting curves of PRRT2-specific primers are shown in Fig. 3A. The relative quantitative PCR results revealed that the CON-iPSCs expressed approximately 16 times more *PRRT2* than the urine cells (Fig. 3B). In addition, the expression of *PRRT2* increased during the induction of the neuroepithelial cell, and peaked in neuroepithelial cells. It decreased when the cells became neurons, and it became smooth thereafter (Fig. 3C). Relative quantitative PCR was performed to clarify if the *PRRT2* mutation will influenced the expression of *PRRT2*. As shown in Fig. 3D, *PRRT2* mRNA was significantly lower in PKD- iPSCs. The above data indicate that PRRT2 may exert physical function in the early phase of the development. Moreover, we found that the induced neurons expressed *PRRT2* at a relative high level, which is consistent with the high expression of *PRRT2* in nervous system. This finding also implied that the induced neuron from PKD-iPSCs was a potential tool to study the function of *PRRT2*.

### Differentiation of PKD-specific glutamatergic neurons

**Fig. 4.**
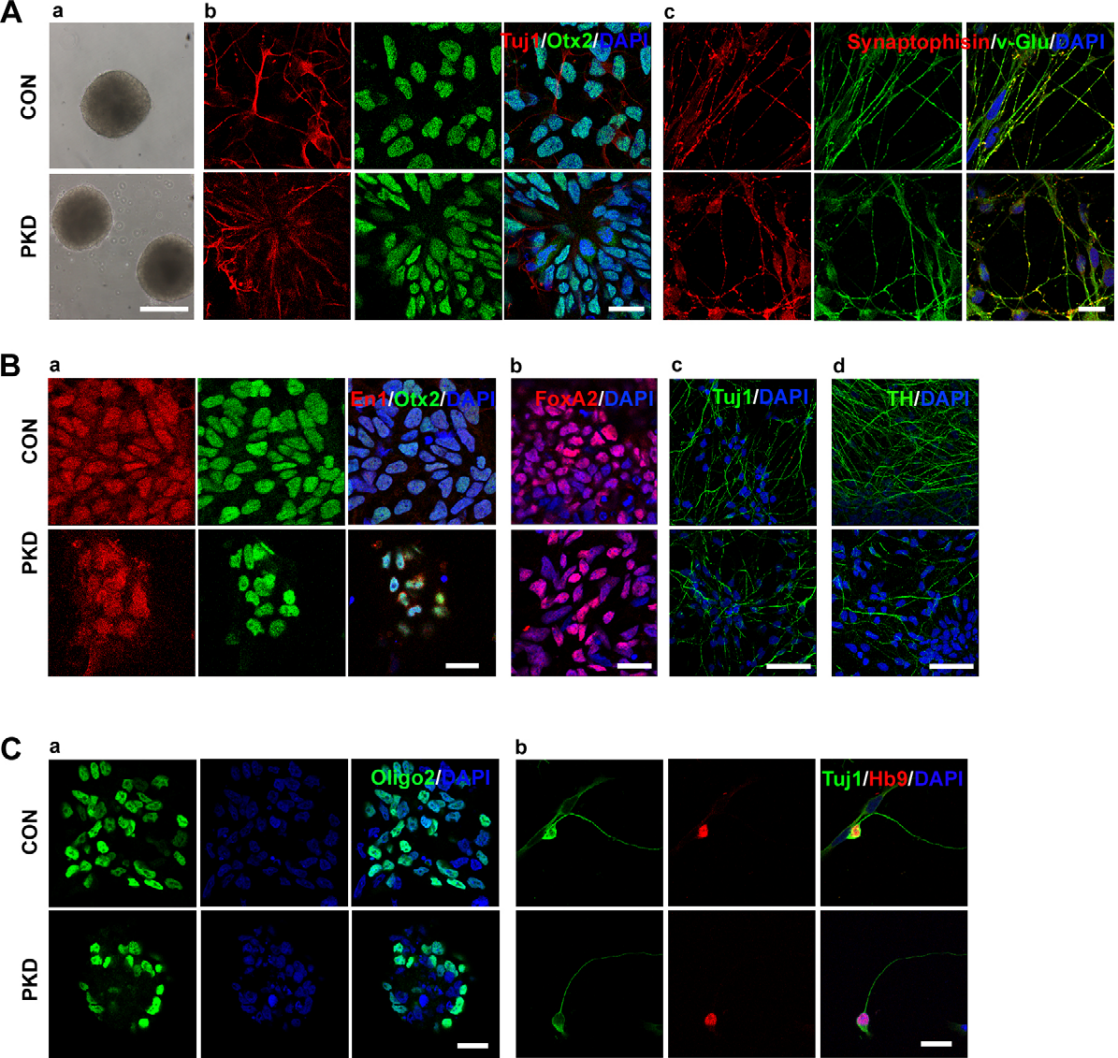
**Urinary cell**-**derived iPSCs differentiated into glutamatergic, dopaminergic and motor neurons.** (Aa) PKD- and CON-iPSCs formed embryo bodies on the 4th day of differentiation. (Ab) On day 28, induced neurons expressed rostral marker Otx2 (green), and Tuj1 (red). (Ac) Cells were positive for Tuj1 and synaptophysin when differentiated for 7 weeks. (Ba) Cells were positive for midbrain dopamine neuron progenitor markers En1 (red), Otx2 (green) and DAPI (blue) on the 24th day. (Bb) Midbrain dopamine neuron progenitor expressed FoxA2 (red) on the 24th day. (Bc,d) Cells expressed mature neurons marker Tuj1 (Bc) and TH (Bd). (Ca) Immunostaining of motor neuron progenitor marker Olig2. (Cb) Neuronal identity of motor neurons is confirmed by co-expression of HB9 (red) and TuJ1 (green) in dissociated patient-specific motor neuron cultures on the 28th day. Scale bars: 200 µm in Aa; 50 µm in Bc,d; 20 µm in Ab,c, Ba,b and C.

To test the differentiation capacity of iPSCs, the floating suspension culture was used to form embryonic bodies (EBs) as previously described (Takahashi et al., 2007). We found that both the PKD-iPSCs and CON-iPSCs could form EBs (Fig. 4Aa). The iPSCs were then induced and differentiated into forebrain glutamatergic neurons using a well-established differentiation protocol (Li et al., 2009). At day 26, the induced neurons expressed neuronal cell markers Tuj1 and MAP2 (data not shown). These cells uniformly exhibited a rostral phenotype, which was confirmed by the expression of the anterior transcription factor Otx2 (Fig. 4Ab). This is in accordance with a previous report (Li et al., 2009). Without the exogenous morphogen, these neurons are predicted to generate glutamatergic neurons of the dorsal cortex (Li et al., 2009), which was verified again in our experiments (Fig. 4Ac). These neurons expressed synaptophysin when they were cultured in the neuronal induction medium for 7 weeks (Fig. 4Ac).

### Generation of PKD-specific DA neurons

We next induced the iPSCs to differentiate into towards DA neurons, using a previously described protocol (Zhang and Zhang, 2010). The differentiated DA neurons induced in this way were mostly of the midbrain origin, as revealed by the co-expression of Otx2 and En1 (Fig. 4Ba**)**. We found that all iPSC lines could generate DA neurons, which was confirmed by the positive staining for FoxA2 (Fig. 4Bb), Tuj1 (Fig. 4Bc) and tyrosine hydroxylase (Fig. 4Bd).

### Generation of PKD-specific motor neurons

During the neurodevelopment, motor neurons are induced by the exposure of rostral neural progenitors to two consecutive signals: retinoic acid (RA) and sonic hedgehog (SHH) (Wichterle et al., 2002). In this study, RA was applied to the primitive neuroepithelial cells at day 10 (Hu and Zhang, 2010). Then, at the same time, recombinant SHH and RA were applied to the definitive neuroepithelial cells for 2 weeks (Hu and Zhang, 2010), which specified the neuroepithelial cells into Olig2-expressing motor neuron progenitors (Fig. 4Ca). By the end of 5 weeks, immunocytochemical staining of these cultures showed that Tuj1-positive neurons co-expressed HB9, which indicated that the obtained cells were precursors of motor neurons (Fig. 4Cb). These cells were maintained in the neuron differentiated medium (NDM) supplemented with BDNF, GDNF, IGF, cAMP, and ascorbic acid (Hu and Zhang, 2010). Thus, patient-specific iPSC lines are capable of direct differentiation into motor neurons, which may be affected in PKD.

### The PKD-iPSC-induced neurons were functional neurons

**Fig. 5.**
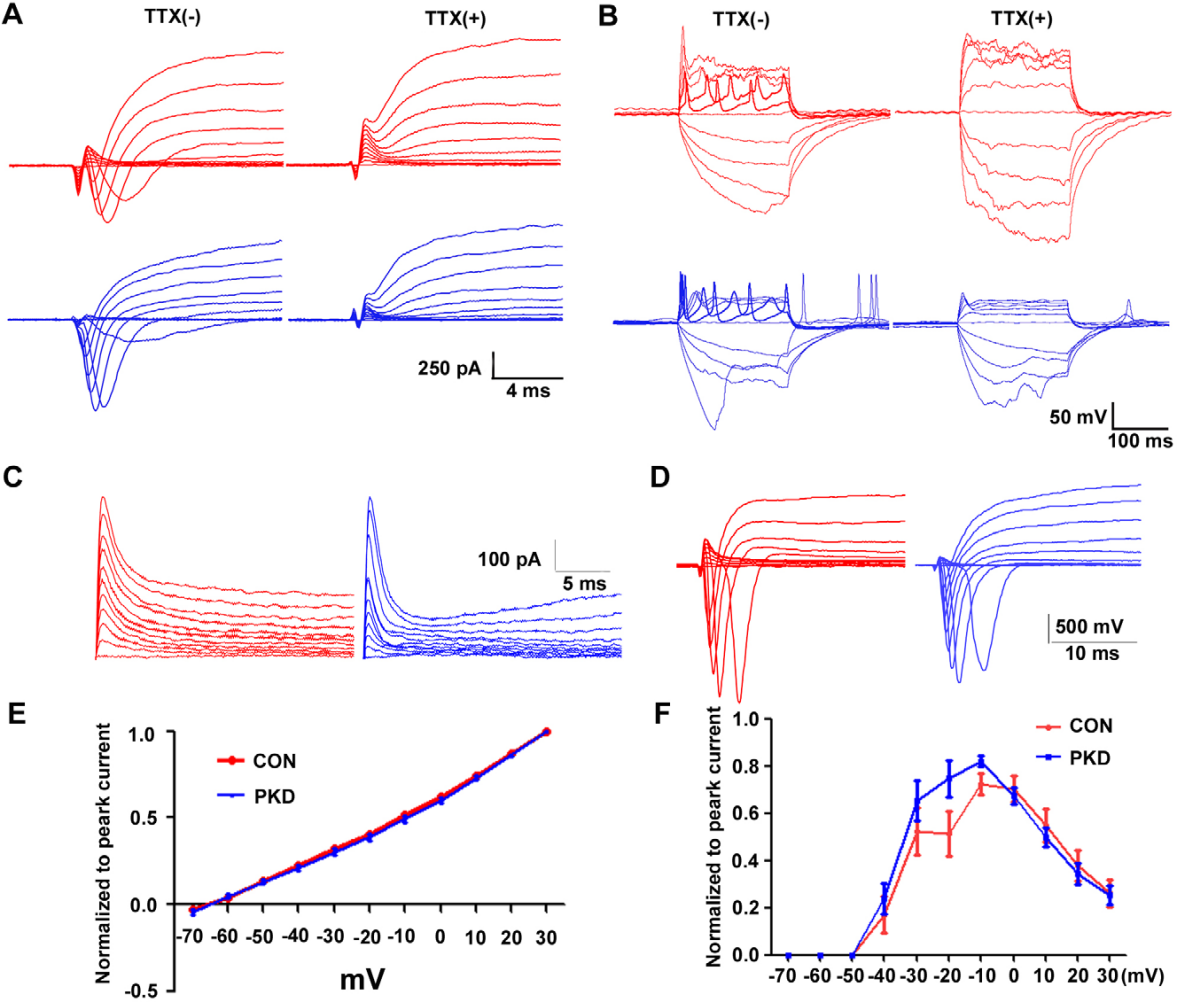
**Electrophysiological properties of PKD-iPSCs derived neurons.** (A) Cells were held at −70 mV, and inward sodium currents were elicited by voltage steps from −70 to +30 mV in 10 mV increments for CON (red) and PKD (blue) iPS derived neurons. (B) Current injection-induced action potential of both CON- and PKD-iPSC-induced neurons. (C) Representative traces show systematic increase in I_A_ generated by the hyperpolarizing prepulses. (D) Both CON- and PKD-iPSC-induced neurons could generate sodium current using the same pulse protocol as in A. (E) Statistical analysis of C. The I_A_ currents showed no differences between CON- and PKD-iPSC-induced neurons when normalized to the peak current. To test whether different path voltage will influence the I_A_ current between the two groups two-way ANOVA was used for the data analysis. (F) Statistical analysis of D. No significant difference was detected of the fast activated and deactivated sodium currents between the CON- and PKD-iPSC-induced neurons. Two-way ANOVA was used for the data analysis. Error bars represent s.e.m.

Because induced neuronal cells can exhibit the morphology of neurons, we probed to see if they have the same capabilities as neurons. Whole cell recording were performed in the CON- and PKD-iPSCs derived neurons. We were able to evoke the voltage dependent potassium currents, and the fast activating and deactivating sodium currents (Fig. 5A). The action potential could be induced in neurons 10 days after being placed on glass cover slips (Fig. 5B). Both the sodium currents and action potentials could be blocked by titrate tetrodotoxin (TTX) treatment (Fig. 5A,B). These data indicated that functional mature neurons could be induced from both CON- and PKD- iPSCs.

### The membrane properties of PKD-iPSC-induced neurons are not different than their healthy counterparts

Because of the clinical similarities between PKD and ion channel disorders, it has been hypothesized that PKD is a channelopathy (Fourcade et al., 2009). We tested the membrane properties of the induced neurons from both CON- and PKD- iPSCs. We found that the voltage gated potassium and sodium channels from PKD-iPSCs and CON-iPSCs derived neurons showed similar activation and inactivation properties (Fig. 5C-F).

## DISCUSSION

In the present study, we generated iPSCs from one PKD patient carrying the c.649dupC mutation by introducing four reprogramming factors (Oct4, Sox2, Klf4 and c-Myc) into cells from the urine sample. The iPSCs exhibited all characteristic properties of pluripotent stem cells and maintained the disease-specific mutation. The mutation did not influence their ESC-like characteristics, but it did decrease the expression of *PRRT2* in the PKD-iPSCs. We analyzed the differentiation potential of the PKD- and CON-iPSCs. Despite of heterogeneity in efficiency, both CON- and PKD-iPSCs could be differentiated into forebrain glutamatergic projection neurons, midbrain dopaminergic neurons, and spinal motor neurons. Typical ion channels currents were recorded in the iPSC-derived neurons, but no difference was detected between the two cell lines. Taken together, the PKD-iPS cells provide a valuable platform to elucidate the functions of PRRT2 and the molecular pathogenesis of PKD.

Efforts to understand the underlying pathology of many neurological diseases such as PKD are hampered by the difficulty in obtaining biopsy materials, patient-derived neural cells or tissues because of the limited accessibility to the brain. PKD, BFIE and ICCA are all benign disorders with favorable outcomes (Heron and Dibbens, 2013). Clinically, it is unnecessary and impossible to biopsy the brain tissues of those patients. Lack of feasible modeling tool deterred the efforts to investigate the potential mechanisms of these disorders. Human iPSCs, characterized by their ability to self-renew and to differentiate into any cell neuronal type, are a powerful tool for modeling diseases, screening drugs and studying regenerative medicine (Allodi and Hedlund, 2014; Isobe et al., 2014; Ruggieri et al., 2014). The generation of various PKD-specific iPSCs may help elucidate the mechanisms of the phenotypic heterogeneity among individuals with the same genetic mutation.

Human iPSCs have been generated with varied efficiencies from multiple tissues, including skin fibroblasts (Takahashi et al., 2007), keratinocytes (Aasen et al., 2008), melanocytes (Utikal et al., 2009), adipose stem cells (Sun et al., 2009; Esteban et al., 2010), and peripheral blood (Loh et al., 2010; Staerk et al., 2010; Seki et al., 2012). However, individuals have to undergo invasive procedures in order to obtain these cells. Several groups have generated human iPSCs from exfoliated renal epithelial cells present in urine (Song et al., 2011; Zhou et al., 2012; Wang et al., 2013). This method is advantageous in many circumstances. The isolation of urinary cells is simple, safe, and fast, and readily accepted by patients.

In the present study, we collected primary urinary cells from a patient carrying the c.649dupC hotspot mutation and reprogrammed the cells into iPSCs. The two iPS cell lines exhibited strong alkaline phosphatase activity (Fig. 1C) and expressed several ES cell-associated antigens (Fig. 1D-H). As previously reported, these cell lines can be expanded without losing their ESC-like characteristics (Takahashi and Yamanaka, 2006; Takahashi et al., 2007) and differentiated into cell types representative of each of the three embryonic germ layers (Fig. 1I). The patient-derived iPSCs could faithfully maintain the patient's specific *PRRT2* mutation (Fig. 2B). All of these results indicated that we successfully converted the urine cells of a PKD patient into iPSCs, thus developing an important resource. In addition, the PKD hotspot mutation did not seem to influence the propagation of patient-derived iPSCs.

Relative quantitative PCR was performed to determine if the iPSCs could be used to study the pathology of PKD and the physiological function of PRRT2. The expression of the *PRRT2* in the iPSCs was significantly increased (Fig. 3B). It continued to increase during the process of neuron induction and reached its highest level at the neuroepithelial stage, which meant that PRRT2 might exert its physiological function at the neuronal progenitor stage. *PRRT2* expression was maintained at a sustained level in neurons (Fig. 3C). The expression pattern of human *PRRT2* during development is similar as that of mice (Chen et al., 2011). In addition, the *PRRT2* mRNA in the PKD-iPSCs was significantly lower (Fig. 3D), which indicates that the hotspot mutation of the PRRT2 may disturb the expression of *PRRT2*. The underlying mechanism of these differences in expression needs further investigation.

Although the gene responsible for PKD has been known for four years, the pathophysiology of PKD remains largely unknown. Brain imaging studies of primary and secondary PKD indicate that the malfunction of cortical-basal ganglia circuit caused by PRRT2 mutations may contribute to the pathology of PKD (Luo et al., 2013). There are many types of neurons in this system, including cholinergic, GABAergic, glutamatergic, dopaminergic, motor, and serotonergic neurons. It was a mystery what type of neurons was affected in PKD. Recent advances in human iPSC technology make it possible to obtain patient- and region-specific functional neural cells. For example, cholinergic (Liu et al., 2013b), GABAergic (Ma et al., 2012; Liu et al., 2013a), glutamatergic (Li et al., 2009), dopaminergic, (Ma et al., 2011) and serotonergic neurons (Shimada et al., 2012; Waschek, 2012) can be differentiated from hiPSCs.

Under serum-free conditions, hiPSCs differentiated into neuroepithelial cells with anterior characteristics (Li et al., 2009), which was verified by the expression of the anterior transcription factors Otx2 (Fig. 4Ab). Without exogenous morphogen, by default, Wnt signaling (Li et al., 2009) promoted the neuroepithelial progenitors into glutamatergic neurons of the dorsal cortex and expressed the vesicular glutamate transporter (V-glut) (Fig. 4Ac). After a 2-week induction period, these anterior precursors can be efficiently patterned into midbrain dopaminergic neurons with FGF8b and SHH (Perrier et al., 2004; Zhang and Zhang, 2010). Midbrain DA neurons could be generated from both PKD-iPSCs and CON-iPSCs, which was confirmed by the expression of FoxA2 (Fig. 4Bb) and TH (Fig. 4Bd) and the co-expression of Otx2 and En1 (Fig. 4Ba). To induce the region-specific spinal motor neurons, rostral neural progenitors were given two consecutive signals: RA and SHH (Li et al., 2005; Hu and Zhang, 2010). After 4 weeks of induction, the Tuj1-positive neurons co-expressed the marker of precursors of motor neurons, HB9 (Fig. 4Cb), which indicates that patient-specific iPSC lines are capable of differentiating into motor neurons. These motor neurons can generate action potentials (Fig. 5B). All these data suggest that the PKD-iPSCs have the potential to differentiate into region-specific neurons and to provide a useful platform for the investigation of PKD pathology.

PKD has been assumed to be a channelopathy. It has been confirmed that *PRRT2* mutations can cause infantile convulsions and paroxysmal dyskinesia alone or in various combinations besides PKD (Guerrini and Mink, 2012). In order to test this hypothesis, whole cell recordings were performed to test the voltage gated sodium and potassium channels. Our results showed that the properties of the fast activating and deactivating sodium channels and I_A_ currents showed no difference between neurons from CON- and PKD-iPSCs. Membrane permeable ion channels in patient-derived iPSCs should be studied to clarify this issue in the future.

To our knowledge, this study is the first to report an iPSC line derived from PKD. The PKD-iPSCs maintained the disease-specific mutation, exhibited all properties of pluripotent stem cells, and differentiated into region specific neurons. Therefore PKD-iPSCs provide a platform for investigating the proposed molecular and cellular mechanisms of PKD, BFIE, and ICCA. In addition, thec.649dupC hotspot mutation may cause PKD by down regulating of *PRRT2* mRNA, and it may be that PKD is not a channelopathy disease.

## MATERIALS AND METHODS

### Isolation and culture of human urinary cells

The urinary cells were collected using a previously established protocol with slight modifications (Zhou et al., 2012). The research ethics committee of Huashan Hospital approved the study protocol, and informed consent was obtained from the donor. Briefly, the mid-stream urine sample was collected from one PKD patient with the *PRRT2* c.649dupC mutation, followed by 150 ***g*** centrifugation to obtain the renal exfoliated cells. The primary urine cells were cultured in renal epithelial cell growth medium (REGM) (Lonza Group Ltd, Basel, Switzerland).

### Reprogramming of urinary cells

Retroviruses carrying human Oct4, Sox2, Klf4 and c-Myc factors were produced in the 293T cell line. Viral supernatant was concentrated by Amicon Ultra Centrifugal Filters (Merck Millipore, Darmstadt, Germany) and filtered through a 0.45-μm cellulose acetate filter before being added to urine cells. A 1:1:1:1 mix of retroviruses was added to primary urinary cells at a multiplicity of infection (MOI) of 10 in the presence of 10 ng/ml polybrene. At 24 h post-transduction, cells were harvested and plated onto X- ray irradiated MEFs (mouse embryo fibroblasts, 5×10^4^ cells per well) in a six-well plate. The next day, the medium was replaced with hESCs culture medium and changed every other day. On the 21st day, iPS colonies were picked and plated onto new culture dishes.

### Cell culture

Human iPSC lines (CON-iPSC-1, CON-iPSC-2, PKD-iPSC-1 and PKD-iPSC-2, passages 20–60) were cultured onto a feeder layer of irradiated embryonic mouse fibroblasts using human ESC growth medium with DMEM/F12, 20% Knockout Serum Replacer, 1×MEM non-essential amino acids solution, 1×GLUTAMAX™ (Invitrogen, New York, USA), 0.1 mM β-Mercaptoethanol (Sigma, St Louis, USA) and 4 ng/ml FGF-2 (Gibco, New York, USA). Cells were passaged using 1 U/ml dispase (Invitrogen) and replated at a dilution of 1:5. CON-iPSC-1 and CON-iPSC-2 were gifts from Professor Wan-Jin Chen at Department of Neurology and Institute of Neurology, First Affiliated Hospital, Fujian Medical University, China.

### Alkaline phosphatase staining

Alkaline phosphatase activity was detected using the Alkaline Phosphatase Staining Kit (Stemgent, Cambridge, USA).

### Teratoma formation and analysis

The urine-derived iPSCs were detached and injected subcutaneously into each flank of non-obese diabetic/severe combined immune deficient (NOD/SCID) mice (approximately 5×10^6^ cells per site). Tumors were collected two months after injection and processed for hematoxylin-eosin (H&E) staining. Animal experiments complied with all relevant institutional and national animal welfare laws, guidelines and policies.

### RNA isolation and relative quantitative PCR

Total mRNA was isolated by guanidinium thiocyanate-phenol-chloroform extraction (Invitrogen). Then 0.5 µg mRNA was used for reverse transcription into cDNA using the PrimeScript™ RT Master Mix (TAKARA, Kyoto, Japan). SYBR Premix Ex Taq™ (TAKARA, Kyoto, Japan) was used for the relative quantitative PCR reaction. The following primers were used for human-*β-actin* (forward 5′-CTC CAT CCT GGC CTC GCT GT-3′ and reverse 5′-GCT GTC ACC TTC ACC GTT CC-3′) and human*-PRRT2* (forward 5′-TCC CCT CTC CCA TCT CAA GA-3′ and reverse 5′-GGG TCT CTG TGG TTT CTG GA-3′).

### Karyotype analysis

On the 4th day of the cell propagation, 2 µg/ml of colchicine was added to the cells for 3 h, and the cells were collected and resuspended in 0.58% (w/v) KCl by adding drops and flicking the tube. After incubation in the 37°C for 10 min, 1/12 fix solution (methanol: glacial acetic acid 1/1) was added to the cell resuspension for 10 min. The cells were centrifuged and mixed in 10 ml of fresh fix solution at room temperature for 30 min. The procedure was repeated for three times, and then, the cells were placed on slides. The slides were treated with 0.025% trypsin in 0.9% NaCl for 15 s and stained in fresh Giemsa stain (Sigma) in 5 mM phosphate buffer (pH 6.8) for 10 min. The karyotype was analyzed with the MetaScan Karyotyping System (IMSTAR SA. Paris, France).

### Short tandem repeats analysis

The genomic DNA was amplified using a GoldenEye™20A STR Multiplex PCR kit (QIAGEN, Dusseldorf, Germany). The ABI Prism3100 Genetic Analyzer (Applied Biosystems, Foster City, USA) was used to analyze the microsatellites and amelogenin alleles.

### Induction of functional neurons

Differentiation of iPSCs into different types of functional neurons was performed according to the previous protocols (Li et al., 2009; Hu and Zhang, 2010; Zhang and Zhang, 2010) but with some modifications (Surmacz et al., 2012). These protocols included five morphological identifiable stages, the ESCs, ESC aggregates, neuroepithelia (NE) in the form of neural tube-like rosettes, neural progenitors in NE aggregates, and post-mitotic neurons.

#### Induction of glutamatergic neurons

For the induction of glutamatergic neurons, iPS colonies were detached and formed embryo body like aggregates (EBs) in hESC medium supplied with SB431542 (Tocris, Taiwan, China) and LDN193189 (Stemgent, Cambridge, USA). After 4 days of suspension culture, the ESC growth medium was replaced with neural induction medium (NIM) with the same compounds added to guide neuroectodermal specification. The EBs were attached onto the culture surface on the 7th day, where columnar neural epithelial cells appeared and organized into rosettes. The procedure lasted for 8 days. On the 15th day, the neural rosettes were enriched by detaching the rosettes. And the neuroepithelial aggregates were expanded in the same medium for another 10 days. Finally, the neuroepithelial aggregates were dissociated and differentiated into nearly pure neurons.

#### Differentiation of dopaminergic neurons

A protocol reported by Zhang and Zhang (2010) was adopted for the differentiation of dopaminergic neurons. The neural tube-like rosettes were maintained in NIM medium supplemented with 50 ng/ml FGF8b (R&D, Minneapolis, USA) and 100 ng/ml SHH (R&D, Minneapolis, USA) from day 10 for one week. On the 17th day, the neuroepithelial progenitors were enriched and expanded in NIM containing FGF8b, SHH, B27 (Invitrogen) and ascorbic acid (Sigma) for another week. Then, the neural progenitor aggregates were dissociated to single cells and plated onto a culture surface pre-coated with laminin using conditioned NDM containing 200 μM ascorbic acid, 1.0 mΜ cAMP (Sigma), 1 ng/ml TGFβ3 (R&D), 10 ng/ml BDNF (R&D), 10 ng/ml GDNF (R&D) and Wnt3a -conditioned medium (1×) for 3 weeks. On the 44th day 44, FGF8b, SHH and Wnt3a conditioned medium was withdrawn and the cells were maintained in NDM with ascorbic acid, cAMP, TGFβ_3_, BDNF and GDNF.

#### Differentiation of motor neurons

For the differentiation of motor neurons, 0.1 μM RA (Sigma) was added to NIM from day 10 to day 15 to pattern the cells to the upper spinal cord phenotypes, as in the previously described protocol described previously (Hu and Zhang, 2010). The enriched neural rosettes were cultured in the same medium supplemented with recombinant SHH at 100 ng/ml and RA at 0.1 μM for another 2 weeks. On the 29th day, the progenitor spheres were plated onto glass coverslips and double-coated with polyornithine and laminin in the NDM supplemented with 10 ng/ml BDNF, 10 ng/ml GDNF, 10 ng/ml IGF1, 1 μM cAMP and 200 ng/ml ascorbic acid.

### Immunocytochemistry

Immunofluorescence on coverslip cultures was performed according to previous reports (Yan et al., 2005). Cells were fixed in 4% paraformaldehyde in PBS for 20 min and blocked in blocking solution (PBS containing 10% normal donkey serum and 0.2% Triton X-100) for 1 h. The following primary antibodies were used: mouse anti-SSEA-3 (1:200), mouse anti-SSEA-4 (1:400), mouse anti-Nkx6.1 (1:100), mouse anti-En1 (1:100) (DSHB, Iowa City, USA), anti-TRA-1-60 (1:150), anti-TRA-1-81 (1:150), mouse anti-Synaptophysin (1:1000), rabbit anti-v-Glut1 (1:2000) (Millipore, Billerica, USA), anti-Nanog (1:150), goat anti-Otx2 (1:500) (R&D), mouse anti-Tuj1 (1:5000) (Abcam, Cambridge, UK), rabbit anti-MAP2 (1:1000), mouse anti-TH (1:1000) (Sigma), mouse anti-HB9 (1:50) and goat anti-FoxA2 (1:500) (Santa Cruz, California, USA). After an overnight incubation at 4°C, species-specific secondary antibodies conjugated with Alexa Fluor 488 or 594 or CY5 (1:1000) (Invitrogen) were applied for 1 h at room temperature. Cell nuclei were stained with DAPI (Sigma). Images were obtained using a Leica TCS SP8 confocal laser-scanning microscope (Leica, Wetzlar, Germany).

### Electrophysiology

Somatic whole-cell patch-clamp recordings using a voltage clamp configuration were performed in PSC-derived neurons at approximately 50 day. Cells selected for electrophysiological recordings had neuron-like shapes. Briefly, neurons were held at −70 mV and steeped to +30 mV from −60 mV in 10 mV increments to record the Na^+^/K^+^ channel activities. The bath solution consisted of 135 mM NaCl, 3 mM KCl, 2 mM CaCl_2_, 1 mM MgCl_2_, 10 mM HEPES, 11 mM glucose, and 10 mM sucrose, pH 7.4 and 290 mOsm. Recording pipettes were filled with an intracellular solution containing 140 mM KCl, 9 mM NaCl, 1 mM MgCl_2_, 10 mM HEPES, 0.2 mM EGTA, 2 mM Mg^2+^-ATP, and 0.25 mM Na^+^-GTP, pH 7.2 and 290 mOsm. Data were analyzed with Clampfit 10.2. For the detection of voltage-dependent potassium A-current (Ia), a well established protocol was used as previously described (Deadwyler et al., 1995). The materials used above were purchased from Sigma.
